# Association of Pathology Markers with Somatostatin Analogue Responsiveness in Acromegaly

**DOI:** 10.1155/2022/8660470

**Published:** 2022-09-26

**Authors:** George Kontogeorgos, Vyron Markussis, Eleni Thodou, Efi Kyrodimou, Theodossia Choreftaki, Panagiotis Nomikos, Kostas I. Lampropoulos, Stylianos Tsagarakis

**Affiliations:** ^1^First Propaedeutic Department of Internal Medicine, Division of Endocrinology, Laikon Hospital, National and Kapodistrian University of Athens, Athens, Greece; ^2^Department of Pathology and Pituitary Tumor Reference Center, “G. Gennimatas” General Hospital of Athens, Athens, Greece; ^3^Independent Research, Athens, Greece; ^4^Department of Pathology, University of Thessaly, Larissa, Greece; ^5^Department of Neurosurgery, “Hygeia” Private Hospital, Athens, Greece; ^6^Department of Gamma Knife Radiosurgery, “Hygeia” Private Hospital, Athens, Greece; ^7^Department of Endocrinology, “Evangelismos” Hospital, Athens, Greece

## Abstract

**Background:**

Somatotroph adenomas (SAs) exhibit a variable responsiveness to somatostatin analogue (SS-a) treatment, a process that is not well understood. We investigated established and novel histological markers as predictors of SS-a responsiveness.

**Methods:**

We retrospectively investigated pathology samples from 36 acromegalic patients that underwent transsphenoidal surgery. Clinical, hormonal, and imaging data were available in 24/36 patients, before and after SS-a treatment. Specimens were semiquantitatively analyzed with immunocytochemistry for Ki-67, KER, SSTR-2, SSTR-5, ZAC-1, E-cadherin, and AIP.

**Results:**

Collectively, 18 (50%) adenomas were each classified as densely/sparsely granulated somatotroph adenomas (DGSAs/SGSAs), respectively. Patients that received preoperative SS-a had lower expression of SSTR-2 compared to those that did not (2.0 (1.0, 3.0) vs. 3.0 (3.0, 3.0), *p* = 0.042). Compared with DGSAs, SGSAs had higher Ki-67 labeling index (LI) (1.0 (0.5, 1.0) vs. 2.0 (1.0, 3.5), *p* = 0.013), and a higher proportion of high MR T2 signal (1 (6%) vs. 6 (33%), *p* = 0.035), and tended to express less ZAC-1 (*p* = 0.061) and E-cadherin (*p* = 0.067). In linear regression corrected for baseline growth hormone (GH), ZAC-1 immunostaining was significantly associated with a decrease in GH levels after SS-a treatment (beta (95% confidence interval): −1.53 (−2.80, −0.26), *p* = 0.021). No markers were associated with changes in circulating insulin-like growth factor-I (IGF-I) after treatment with SS-a.

**Conclusion:**

The novel marker ZAC-1 was associated with GH response to medical treatment with SS-a. The SGSA cases were characterized by higher Ki-67 values and MR T2 signals indicative of an inferior response to SS-a. These findings improve our understanding of the mechanisms underlying SA response to medical treatment.

## 1. Introduction

Acromegaly is a systemic chronic endocrine disease that results from growth hormone (GH) hypersecretion and often requires multimodal management [1]. In the substantial majority of cases, the source of GH hypersecretion is a somatotroph adenoma (SA). Treatment of acromegaly with somatostatin analogues (SS-a) results in reduction of circulating GH and insulin-like growth factor-I (IGF-I), and also significant tumor size reduction in a substantial proportion of patients [2, 3]. Indeed, in unselected patients with acromegaly, the success rate of therapy with SS-a, evaluated using composite GH and IGF-I values, is approximately 60–70% [3, 4]. However, this proportion varies widely between different studies, and *a priori* selection of optimal responders to specific treatment modalities remains elusive [2, 5, 6]. Importantly, early identification of nonresponders to SS-a would facilitate the prompt selection of alternative treatment modalities.

Since response to treatment is not universal, several factors have been investigated as potential indicators of resistance to SS-a treatment. Among others, germline mutations in the aryl hydrocarbon receptor-interacting protein (AIP) gene [7] and alpha stimulating activity polypeptide 1 (GNAS or GSP) have been implicated in this setting [8]. Furthermore, previous studies have proposed the use of somatostatin receptor (SSTR) expression in SAs, mostly SSTR-2 and SSTR-5, as a predictive marker for treatment response to SS-a. Nevertheless, the current literature in the field is limited to specific evaluation of individual markers or small panel of markers. To our knowledge, a single unifying head-to-head comparison of multiple histological markers for the evaluation of resistance to SS-a treatment, has as of yet not been performed.

We thus hypothesized that by simultaneously studying established and novel pathology markers we would identify histologic profiles that could optimally predict treatment response to SS-a (hormonal and tumor shrinkage) in patients with SAs. For this reason, in addition to the status of SSTR-2 and SSTR-5, we studied the Ki-67 labeling index and the expression of the novel markers E-cadherin, ZAC-1, and aryl hydrocarbon receptor-interacting protein (AIP).

## 2. Material and Methods

### 2.1. Cases

This retrospective, noninterventional, single-center study included acromegalic patients that underwent transsphenoidal surgery at “Hygeia” hospital (Athens, Greece). Frozen pathology samples from these patients were further investigated immunohistochemically at the Pituitary Reference Center of “Georgios Gennimatas” General Hospital (Athens, Greece). The protocol was approved by the “Hygeia” Hospital Medical Ethics Committee (approval number: 598/15.04.2015), and informed consent was obtained from all participants before inclusion to this study. The condition for being included in the study was the presence of sufficient and well-preserved tissue of specimens, stored in paraffin blocks. Out of 113 consecutive patients that underwent surgery between 2004 and 2018, a total of 36 had specimens that were of sufficient quality to warrant inclusion in the study. Of those, 25/36 patients received treatment with somatostatin analogue (octreotide or lanreotide) for at least 3 months, either preoperatively or postoperatively.

All adenomas were initially diagnosed and classified by histology and pituitary hormone immunohistochemistry, including growth hormone (GH), prolactin (PRL), adrenocorticotropic hormone (ACTH), *β*-subunits of thyroid stimulating hormone (TSH), follicle stimulating hormone (FSH), luteotropic hormone (LH), and the *α*-subunit of glycoprotein hormones. The initial diagnostic panel also included the Ki-67 proliferation marker and low molecular weight cytokeratins (CAM 5.2).

### 2.2. Adenoma Subtypes

According to the cytokeratin immunohistochemical pattern, SAs were separated in densely granulated (DGSA) and sparsely granulated (SGSA). By histology, SGSAs demonstrate a round cytoplasmic inclusion, known as a fibrous body. DGSAs show a diffuse cytoplasmic distribution of low molecular weight cytokeratins, whereas in SGSAs, the immunoreactivity is restricted to the fibrous body. Three adenomas showing both patterns of cytokeratin distribution, known as transitional, were grouped together with DGSAs [9].

### 2.3. Immunohistochemical Protocols

**Figure 1:**
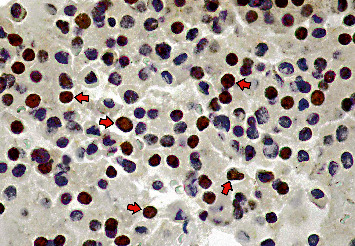
Sparsely granulated somatotroph adenoma with strong and extensive nuclear reactivity for ZAC-1, indicated with illustrative arrows (Immunohistochemistry, Hematoxyline-DAB, 40X).

For the present study, additional sections from the tissue blocks were immunostained for SSTR-2 and SSTR-5, E-cadherin, aryl hydrocarbon receptor-interacting protein (AIP), and zinc-finger protein (ZAC-1). A representative histological section of strong nuclear ZAC-1 immunoreactivity is presented in Figure 1. All tissue specimens were fixed in 10% buffered formaldehyde and embedded in paraffin. Tissue sections of 5 *μ*m were cut from paraffin blocks and attached to positively charged glass slides. To detect the antigen-antibody biding cites, application of primary FLEX target retrieval solution (high *pH*) followed by the one-step Envision polymer detection system (Dako A/S, Glostrup, Denmark) was used as a secondary link to DAB chromogen (Sigma, St Lewis, MO, USA). In a few cases, due to repeated sectioning, the tissue material was no longer available for further immunostaining. For SSTR immunohistochemistry, the monoclonal antibodies SSTR-2A, clone UMB-1 (dilution 1 : 1000; Abcam Cambridge, MA, USA), SSTR-5, and clone UMB-4 were applied (dilution 1 : 500; Abcam Cambridge, MA, USA). For demonstration of E-cadherin, a monoclonal antibody, clone HECD-1 (dilution 1 : 100, Abcam Cambridge, MA, USA) was applied. For AIP, a monoclonal antibody, clone 35-2, raised against ARA9 epitope (dilution 1 : 600, Novus Biologicals, Littleton, CO, USA) was used. For demonstration of ZAC-1, a monoclonal antibody, raised against amino acids 211–510 of ZAC-1 of mouse (dilution 1 : 100, Dallas TX, US), was applied.

### 2.4. Immunohistochemical Evaluation

For evaluation of SSTRs, a four-scale scoring system considering the staining intensity and the pattern of membranous distribution of immunostaining was used (Supplementary Table 1) as described previously [10, 11]. Cases with <10% positivity were considered negative. The same scoring system was applied to evaluate membranous immunostaining of E-cadherin. Cytoplasmic immunoreactivity for SSTR was ignored if it depicted the inactive internalized receptor. For assessment of ZAC-1 and AIP immunohistochemistry, a combined double scoring system, similar to the one used for the estimation of estrogen and progesterone receptors, was applied [12]. Accordingly, the number of positive nuclei of 500–1000 cells was counted from “hot spot” areas. The score was based on the semiquantitative estimate of the percentage of positive nuclei and on the intensity of the immunostaining. The final score was determined by multiplying the percentage score of positive cells by the staining intensity score of immunohistochemistry, divided by 2 (Supplementary Table 2). Scores of 1–3 are considered positive.

### 2.5. Statistical Analysis

Statistical analyses were carried out using Stata v. 16 SE (StataCorp. 2019. Stata Statistical Software: Release 16. College Station, TX: StataCorp LLC). Normality of continuous variables was determined by visual inspection of Q-Q plots and/or histograms. Normally distributed variables are presented as mean (standard deviation), continuous not-normally distributed and ordinal variables are presented as median (interquartile range), and binary/categorical variables are presented as numbers (percentage). Between-group comparisons were performed using two-sample *t*-tests for continuous variables, Mann–Whitney *U* tests for continuous not-normally distributed and ordinal variables, and Chi-square tests for binary/categorical variables. The relationship between histological markers and MR measurements as the well as hormonal response was determined using linear or logistic regression analyses where appropriate. For analyses evaluating the change in hormonal levels, correction for baseline hormonal levels was performed in the corresponding regression models. Statistical significance was considered for *p* ≤ 0.05; no adjustment for multiple statistical testing was performed in line with the exploratory nature of this study.

## 3. Results

In 36 cases, the histological specimens were well preserved and sufficient for analysis. From these, 23 (63.89%) were from patients who received SS-a preoperatively. SS-a have been administered preoperatively routinely, while the patients were waiting for their operation appointment. Postoperative SS-a was administered in 2 (5.56%) patients, while the remaining 11 (30.56%) did not receive any medical treatment for acromegaly. Out of the 25 patients that received SS-a, GH/IGF-I and tumor size response to medical treatment was available in 24 patients. Radiotherapy was administered postoperatively and the only study subject that received SS-a postoperatively had not received radiotherapy. Overall, from the 36 cases of the study group, 18 were classified as DGSAs (including 3 with both cytokeratin patterns) and the remaining 18 as SGSAs. The Four Score Scale of immunohistochemical evaluation of all markers used is presented in Supplementary Table 3. Six SGSAs showed a Ki-67 LI value 3% or more (range 3%–5%).

Baseline characteristics for the entire cohort and patients stratified according to adenoma granulation are presented in Table 1. The 36 patients were aged 48.1 ± 11.7 years, and 17 (47%) were female, with 30 (83%) having a macroadenoma. The median adenoma diameter was 16 (12.0, 21.0) mm. Median GH and IGF-1 levels at baseline were 7.85 (5.25, 25.4) *μ*g/L and 856.5 (606.5, 1336.5) ng/mL, respectively. Stratification of baseline characteristics according to tumor granulation into patients with SGSAs and DGSAs demonstrated a higher prevalence of a high *T*2 signal intensity in patients with SGSAs (1 (6%) vs. 6 (33%), *p* = 0.035). In addition, compared with DGSAs, SGSAs had a significantly higher Ki-67 labeling index (1.0 (0.5, 1.0) vs. 2.0 (1.0, 3.5), *p* = 0.013) and to express less ZAC-1 and E-cadherin, although this did not reach statistical significance (*p* = 0.061 and 0.067), respectively.

Patients that received SS-a treatment preoperatively had a lower expression of SSTR2 (2.0 (1.0, 3.0) vs. 3.0 (3.0, 3.0), *p* = 0.042). Linear regression for predicting change in GH (delta GH) before and after treatment with SS-a, based on histological markers, corrected for baseline GH values showed that ZAC-1 staining was significantly associated with a decrease in growth hormone levels (beta (95% confidence interval): −1.53 (−2.80, −0.26), *p* = 0.021) (Table 2). No histological markers were associated with changes in circulating insulin-like growth factor-1 before and after treatment with SS-a (Table 2).

Regression analyses did not reveal any relationship between histological markers and adenoma characteristics on brain MRI. Linear regression analysis corrected for having received SS-a treatment did not demonstrate any association between histologic indices and baseline GH or IGF-1 levels.

## 4. Discussion

In this retrospective study, a variety of established and novel immunohistochemical markers were concomitantly assessed in order to obtain further insight on the biology and medical treatment responsiveness in a cohort of acromegalic patients undergoing transsphenoidal surgery. In good agreement with previous reports, SGSAs were characterized by higher Ki-67 values and MR T2 signal intensity compared to DGSAs. SSTR-2, SSTR-5, and AIP expression did not differ significantly between groups, while ZAC-1 and E-cadherin showed a trend of a higher expression in the DGSA group. Patients that received preoperative treatment with SS-a had a significantly lower expression of SSTR-2. A novel finding in this study was the significant association detected between ZAC-1 expression and the lowering of GH levels following SS-a therapy. The main strength of the study is that all pathology markers were concomitantly used in all available specimens and analyzed using a well-validated scoring system by two experienced pathologists.

Treatment of SAs with first generation SS-a is the first-line option for patients with acromegaly, particularly when surgery fails to control the disease. However, previous studies have reported varying effectiveness of medical treatment in such cases [6]. Prediction of treatment response may thus have therapeutic implications for these patients. Various histological tissue markers have been shown to correlate with responsiveness to SS-a therapy. The most extensively investigated and validated immunohistochemical markers include the granulation pattern, assessed by cytokeratin distribution, SSTR expression, and the Ki-67 LI. As stated previously, according to the immunohistochemical pattern of low molecular weight keratins, SAs are separated in DGSAs and SGSAs [9]. The majority of DGSAs respond better to the administration SS-a compared to SGSAs [13, 14]. In fact, in the present study, SGSAs had a significantly higher Ki-67 LI, and a much higher MR T2 signal intensity compared to DGSAs implicating an inferior responsiveness to SS-a therapy considering the previously reported role of Ki-67 in this context. Namely, Ki-67 has been identified as a predictor of treatment response with octreotide LAR, independent of the SSTR2 status. In addition, Ki-67 was associated with the adenoma cytokeratin pattern, with a higher Ki-67 LI in SGSAs than in DGSAs [15]. The granulation pattern can also influence *T*2-weighted MR imaging [16]. In line with this observation, a hyperintense signal on T2-weighted imaging has been associated in several studies with a poorer response to SS-a and with the SG pattern in immunohistochemistry [16, 17].

In this cohort, however, GH and IGF-I responses to SS-a therapy did not differ between these 2 adenoma subtypes. Regardless of the histological subtype, SSTR-2 expression is significantly associated with response to treatment with SS-a [18]. In this cohort, both SSTR-2 and SSTR-5 did not differ significantly between SGSAs and DGSAs, and this may explain the lack of a different response to SS-a treatment. In addition, as reported previously, patients who received preoperative treatment with SS-a had a lower expression of SSTR-2 post-treatment [19, 20]. This finding may represent a downregulation of SSTR receptors during treatment as has been proposed elsewhere [21, 22]. It should be noted that patients with negative or cytoplasmic only SSTR-2 expression (scores 0-1) are not responsive to SS-a [23]. This is due to the fact that in contrast to the membrane receptor part representing the signaling element, the internalized SSTR in the cytoplasm is inactive [24].

Cell-to-cell adhesion and polarity are fundamental for adenohypophysial cells. E-cadherin, a typical transmembrane adhesion molecule for epithelial cells, provides a physical link to adjacent cells and also maintains the intracellular cytoskeleton. The extracellular domain of E-cadherin binds adjacent cells together, whereas the intracellular domain of the cytoskeleton is linked to actin through a protein complex with catenins [25, 26]. Variable reduction of E-cadherin expression has also been demonstrated in SAs, particularly in SGSAs [27]. In accordance with this finding, SGSA cases in our study had a tendency to express less E-cadherin; however, this finding did not reach statistical significance. In line with this, another retrospective study demonstrated that E-cadherin was the best molecular predictor of response to SS-a [28].

Other factors implicated in the response to therapy include ZAC-1 and AIP. ZAC-1, a zinc-finger protein, is a tumor-suppressor gene that is downregulated in various tumors including the SAs. The antiproliferative effect of ZAC-1 involves apoptosis and G1 cell-cycle arrest leading to tumor shrinkage [29]. ZAC-1 shows high expression in normal pituitary cells and reduced expression in pituitary adenomas [30]. Somatotroph adenomas, mainly from patients preoperatively treated with SS-a, show high ZAC-1 expression [29, 31]. This finding suggests that ZAC-1 may be downstream of somatostatin signaling in pituitary cells [32]. In fact, a significant positive correlation was found between strong ZAC-1 immunoreactivity and IGF-I, but not GH normalization in a previous study [31]. Furthermore, in our study, ZAC-1 staining was significantly associated with a decrease in GH levels after treatment with SS-a. This finding provides additional support to the notion that ZAC-1 expression might increase after administration of SS-a; thus, it could be used as a histological surrogate of SA responsiveness to SS-a. Notably, in our study, SGSAs had a tendency to express less ZAC-1, although this finding did not reach significance, potentially suggesting that the low response rate to SS-a observed in acromegalic patients with this tumor subtype could be related to a lower overall expression of ZAC-1. This finding will however require independent validation in future studies.

Although both clinical and experimental studies support the existence of a potential link between first generation SS-a and AIP/ZAC-1 expression [33]; in this study, no association was detected between AIP expression and changes in GH or IGF-I levels following SS-a treatment. Moreover, despite previous reports demonstrating a lower expression of AIP in SGSAs and a positive association of AIP with SSTR-2 expression, these findings were not corroborated in our cohort. In fact, AIP was highly and similarly expressed in both DGSAs and SGSAs. In agreement with our findings, poor association between AIP and SS-a response, mostly in AIP-mutated patients, has also been reported previously [7, 33, 34]. One explanation for these discrepant results is that they may merely reflect methodological difficulties in the immunohistochemical detection, interpretation, and calculation of AIP expression [24].

The findings of our study also have potential implications for the selection of individualized therapeutic tracks in patients with SAs. Partly because of their histologic heterogeneity, SAs exhibit a heterogeneous response to treatment with first-line SS-a, necessitating a “trial-and-error” strategy to select patients that might better respond to second-line treatment [28]. A method of patient preselection would both save crucial treatment time, as well as reduce costs associated with ineffective treatments, while also providing the opportunity to generate a personalized treatment pathway for each patient. Different approaches have been taken to resolve this issue, but they have not yet been introduced into clinical practice guidelines [17]. Thus, our finding suggests that ZAC-1 is related to GH's response to SS-a, which could serve as a histologic index that can be used to tailor subsequent therapeutic decision-making, after surgical treatment failure in patients with acromegaly.

### 4.1. Limitations

Our study has several limitations. The study is by design retrospective and only a part of the initially available tissue material was of adequate quality to be included in the study. Response data to medical treatment with SS-a were not available in all cases, thus limiting the study cohort further. As such, some analyses may have been underpowered due to the relatively small cohort size. Lastly, since all patients were recruited from a tertiary referral neurosurgery center, a referral bias for including patients with macroadenomas not well controlled with SS-a cannot be excluded.

## 5. Conclusion

In conclusion, in this clinicopathological study with SA specimens, the novel histological marker ZAC-1 was associated with GH response to medical treatment with SS-a. The SGSA cases were characterized by higher Ki-67 values and MR T2 signals indicative of a poor response to SS-a. These findings improve our understanding of SA biology and provide additional tools for the prediction of response to medical therapy.

## Figures and Tables

**Table 1:** Baseline characteristics and statistical comparisons within the entire intervention group, and stratified by sparse or dense tumor granulation. ^*∗*^*p* ≤ 0.05.

Variable	Whole group	Densely granulated	Sparsely granulated	*p*-value
Group size	36	18	18	N/A
Age	48.1 (11.7)	50.6 (11.0)	45.6 (12.2)	0.20
Female sex	17 (47%)	7 (39%)	10 (56%)	0.32
Radiotherapy	6 (17%)	1 (6%)	5 (28%)	0.074
Macroadenoma	30 (83%)	15 (83%)	15 (83%)	0.99
Suprasellar extension abutting the optic chiasm	14 (39%)	8 (44%)	6 (33%)	0.49
Cavernous sinus invasion	18 (51%)	9 (53%)	9 (50%)	0.86
Sphenoid sinus invasion	6 (17%)	3 (17%)	3 (17%)	0.99
High MR T2-Signal	7 (19%)	1 (6%)	6 (33%)	**0.035** ^*∗*^
Maximal initial diameter (mm)	16 (12.0, 21.0)	15.0 (11.0, 20.0)	16.0 (12.0, 24.0)	0.65
GH before treatment (*μ*g/mL)	7.85 (5.25, 25.4)	13.3 (5.8, 26.8)	7.3 (4.8, 24.0)	0.41
IGF-1 before treatment (ng/mL)	856.5 (606.5, 1336.5)	961.0 (705.0, 1500.0)	851.5 (583.0, 955.0)	0.22
Ki-67	1.0 (0.5, 2.0)	1.0 (0.5, 1.0)	2.0 (1.0, 3.5)	**0.013** ^*∗*^
SSTR-2	3.0 (1.0, 3.0)	3.0 (1.0, 3.0)	2.5 (1.0, 3.0)	0.29
SSTR-5	1.0 (0.0, 3.0)	1.0 (0.0, 3.0)	1.0 (1.0, 3.0)	0.80
ZAC-1	0.5 (0.0, 2.0)	1.0 (0.0, 2.0)	0.0 (0.0, 1.0)	0.061
E-cadherin	1.0 (0.0, 2.0)	2.0 (1.0, 3.0)	0.0 (0.0, 2.0)	0.067
AIP	3.0 (3.0, 3.0)	3.0 (3.0, 3.0)	3.0 (2.0, 3.0)	0.25

**Table 2:** Linear regression for predicting change in GH (delta GH) [first two panels], and IGF-1 (delta IGF-1) [last 2 panels], before and after treatment with SS-a based on histological markers, corrected for baseline GH and IGF-1 values respectively.

Variable	Change in GH		Change in IGF-1	
	Beta (95% CI)	*p*-value	Beta (95% CI)	*p*-value
Ki-67	0.17 (−0.97, 1.31)	0.757	17.15 (−75.75, 110.05)	0.704
SSTR-2	−0.82 (−1.93, 0.28)	0.136	−38.51 (−132.52, 55.50)	0.404
SSTR-5	−0.17 (−1.11, 1.46)	0.783	23.06 (−82.28, 128.40)	0.653
ZAC-1	−1.53 (−2.80, −0.26)	**0.021 ** ^*∗*^	91.15 (−20.17, 202.49)	0.103
E-cadherin	0.27 (−0.98, 1.53)	0.652	7.75 (−95.16, 110.66)	0.877
AIP	0.02 (−2.34, 2.39)	0.985	132.65 (−106.12, 371.41)	0.260
Granulation	1.85 (−0.99, 4.69)	0.189	109.90 (−95.03, 314.83)	0.277

## Data Availability

The data that support the findings of this study are available from the corresponding author upon reasonable request.

## Supplementary Materials

Supplementary Materials Supplementary Table 1. The scoring system, according to the distribution pattern and intensity and immunoreactivity on the cell membrane, was used for SSTR and e-cadherin. Supplementary Table 2. Estimation of the score used for ZAC-1 and AIP immunohistochemistry. Supplementary Table 3. The Four Score Scale of immunohistochemical evaluation of the markers in all cases with available tissue material and in cases pretreated with SS-a.
