# Supplementary material for: Association of Pathology Markers with Somatostatin Analogue Responsiveness in Acromegaly
Source: Int J Endocrinol. 2022 Sep 26;2022:8660470. doi: 10.1155/2022/8660470 (PMC9529452; doi:10.1155/2022/8660470)
Supplement: Supplementary Materials — Supplementary Table 1. The scoring system, according to the distribution pattern and intensity and immunoreactivity on the cell membrane, was used for SSTR and e-cadherin. Supplementary Table 2. Estimation of the score used for ZAC-1 and AIP immunohistochemistry. Supplementary Table 3. The Four Score Scale of immunohistochemical evaluation of the markers in all cases with available tissue material and in cases pretreated with SS-a. [file 8660470.f1.docx]

**Supplementary Table 1**. Scoring system, according to the distribution pattern and intensity and immunoreactivity on the cell membrane, used for SSTR and e-cadherin.

| **Grade** | **Result** | **Cell number** | **Cell membrane pattern**  **and staining intensity** |
| --- | --- | --- | --- |
| 0 | Negative or positive | <10% | Any |
| 1+ | Positive | >10% | Weak, incomplete |
| 2+ | Positive | >10% | Moderate, focally complete |
| 3+ | Positive | >10% | Strong, complete linear |

**Supplementary Table 2**. Estimation of score used for ZAC-1 and AIP immunohistochemistry.

| \| **System A**  **Positive Cells % Proportion Score** \| **System B**  **Intensity Staining Score** \| \| --- \| --- \| \| \| 0 - <1 \| 0 \| Any \| 0 \| \| --- \| --- \| --- \| --- \| \| 1-33 \| 1 \| Weak, incomplete \| 1 \| \| 34–66 \| 2 \| Moderate, focally complete \| 2 \| \| > 67 \| 3 \| Strong, complete linear \| 3 \| \|  \|  \|  \|  \| \| \| \| |  | |
| --- | --- | --- | --- | --- | --- | --- | --- | --- | --- | --- | --- | --- | --- | --- | --- | --- | --- | --- | --- | --- | --- | --- | --- | --- | --- | --- | --- |
|  |  |  |

*The two scores are added together and divided by 2, for a final score with 4 possible values.*

**Supplementary Table 3**. Four score scale of immunohistochemical evaluation of the markers in all cases with available tissue material.

|  | **SCORE** | **SSTR-2** | **SSTR-5** | **e-Cadh** | **ZAC1** | **AIP** |
| --- | --- | --- | --- | --- | --- | --- |
| **DENSELY GRANULATED** | **0** | **1** | **5** | **4** | **6** | **0** |
|  | **1** | **4** | **5** | **4** | **3** | **1** |
|  | **2** | **1** | **2** | **5** | **5** | **1** |
|  | **3** | **12** | **5** | **5** | **3** | **15** |
|  | **SCORE** |  |  |  |  |  |
| **SPARSELY GRANULATED** | **0** | **4** | **4** | **10** | **11** | **1** |
|  | **1** | **2** | **6** | **1** | **3** | **1** |
|  | **2** | **3** | **3** | **2** | **2** | **3** |
|  | **3** | **9** | **5** | **3** | **1** | **13** |

Four score scale of immunohistochemical evaluation of the makers in cases pre-treated with SS-a

|  | **SCORE** | **SSTR-2** | **SSTR-5** | **e-Cadh** | **ZAC1** | **AIP** |
| --- | --- | --- | --- | --- | --- | --- |
| **DENSELY GRANULATED** | **0** | **1** | **3** | **3** | **4** | **0** |
|  | **1** | **3** | **4** | **2** | **0** | **0** |
|  | **2** | **1** | **0** | **4** | **4** | **1** |
|  | **3** | **6** | **3** | **2** | **2** | **9** |
|  | **SCORE** |  |  |  |  |  |
| **SPARSELY GRANULATED** | **0** | **4** | **3** | **6** | **8** | **0** |
|  | **1** | **2** | **5** | **1** | **2** | **1** |
|  | **2** | **1** | **1** | **2** | **2** | **2** |
|  | **3** | **6** | **4** | **3** | **0** | **10** |
